# Bisphenol-A disrupts mitochondrial functionality leading to senescence and apoptosis in human amniotic mesenchymal stromal cells

**DOI:** 10.1038/s41420-025-02620-8

**Published:** 2025-07-16

**Authors:** Sara Ficai, Andrea Papait, Marta Magatti, Alice Masserdotti, Michael Gasik, Antonietta Rosa Silini, Ornella Parolini

**Affiliations:** 1 Department of Life Science and Public Health, Università Cattolica del Sacro Cuore, Rome, Italy; 2 Fondazione Policlinico Universitario “Agostino Gemelli” IRCCS, Rome, Italy; 3 Centro di Ricerca E. Menni, Fondazione Poliambulanza Istituto Ospedaliero, Brescia, Italy; 4 Seqvera Ltd., Helsinki, Finland

**Keywords:** Cell biology, Molecular biology

## Abstract

In today’s context, microplastic pollution has become an increasingly pressing issue not only for the environmental fallout but also for the assumed negative effects on human health. It is now well-established that microplastics (>1 mm in size) can enter the human body through ingestion, inhalation, dermal contact and also maternal-fetal transmission. Alarming were the recent findings of microplastics within the human term placenta. Among the degradation by-products of microplastics, Bisphenol-A (BPA) has emerged as a hazardous chemical, with potential toxicity at multisystemic level, particularly on the earliest stages of human development. Based on these findings, our study focuses on assessing the impact of BPA on properties and functions of mesenchymal stromal cells isolated from the amniotic membrane (hAMSC) of the human term placenta. The amniotic membrane surrounds the fetus, playing a fundamental protective role toward toxic chemicals and pollutants that the mother may encounter. Our research revealed how exposure to increasing concentrations of BPA compromise mitochondrial functionality in hAMSC, resulting in enhanced production of reactive oxygen species at mitochondrial level (mtROS). This, in turn, leads to the stabilization of p53, which triggers an increased expression of p21 and p27 encoding genes and an imbalance in the genetic expression of Bax and Bcl-2. Additionally, we observed upregulated expression of cytokines and chemokines associated with the senescence-associated secretory phenotype (SASP). The increased oxidative stress, which plays a central role in BPA-mediated toxicity, can trigger the activation of the senescence pathways, or culminate in cell death, due to the overwhelming stress conditions. Therefore, our results provide novel insights into the mechanism of action of BPA and elucidates its impact on the functionality of hAMSC. This underscores the pressing need to reconsider the use of BPA as a plastic additive, mitigating the potential adverse effects on babies.

## Introduction

Plastic is extensively used in modern society, and its large-scale production presents a major global challenge [1]. Poor waste management practices, with only 9% of plastics being properly recycled worldwide [2], contribute to the widespread dispersion of plastic materials in the ecosystem. These plastics undergo physical and chemical degradation, breaking down into smaller fragments [1, 3]. Among them, microplastics (>1 mm in size) [4] have attracted considerable attention due to their persistence and potential harm to ecosystems and human health [5].

Human microplastic exposure primarily occurs through ingestion, followed by inhalation and skin contact. Once inside the body, these microplastics can exhibit toxicity at a multisystemic level [5], and there is a growing concern of their safety issued from the point of view of children’s health [6].

Microplastics toxicity is not solely attributed to the possible physical obstruction within organs but can also be influenced by the presence of additives, incorporated during plastic manufacturing, aimed at enhancing its properties [7]. Among these additives, Bisphenol-A (BPA) is one of the most investigated, especially for its detrimental effects on the endocrine and reproductive systems [8, 9]. Studies have emphasized the pervasive nature of BPA exposure across different body tissues, and BPA was identified in human saliva, blood, breast milk, feces, lungs, liver, and placenta [10]. Particularly, the recent discovery of BPA within the placenta has raised significant concerns about its potential impacts on human reproduction and gestation [11]. Specifically, human exposure to BPA during pregnancy has been correlated with obstetric complications such as preeclampsia, fetal growth restriction, miscarriage, and premature birth [12–15].

Despite the growing interest in BPA and its potential health effects, our understanding of how it specifically affects the functionality of the placenta, and consequently fetal development, remains incomplete. Recently, exposure to low doses of BPA during gestation and lactation was shown to be associated with an increased level of pro-inflammatory lymphocytes Th17 in mouse offspring [16]. Furthermore, BPA has been linked to pathological changes in the placenta by interfering with its metabolic functions [17].

While few studies have investigated this aspect using trophoblast cells [17–21] significant knowledge gaps persist regarding its impact on other placental cell types.

Of note, microplastics have been specifically found in the amniotic membrane of the human term placenta [11], and considering the growing evidence that mesenchymal stromal cells (MSC) in placental tissues have a prominent role in generating a functional microenvironment critical to a successful pregnancy [22, 23], it is imperative to understand the effects of BPA exposure on MSC from the amniotic membrane (hAMSC), which is the innermost membrane closest to the fetus. This will be essential to determine if BPA could negatively impact the ability of hAMSC to maintain a functional environment during pregnancy.

Thus, the aim of our study was to investigate, for the first time, the influence of BPA on hAMSC.

Our findings demonstrate that BPA negatively affects hAMSC viability by altering mitochondrial functionality. This alteration is associated with increased production of ROS, which in turn affect the formation of the inflammasome complex and induce senescence. These results offer significant insights into the mechanism of action of BPA on mesenchymal stromal cells, providing new information on its implications for amniotic membrane functionality during fetal development.

## Results

### Increasing concentrations of BPA reduce hAMSC viability and trigger an impairment in mitochondrial functionality

**Fig. 1:**
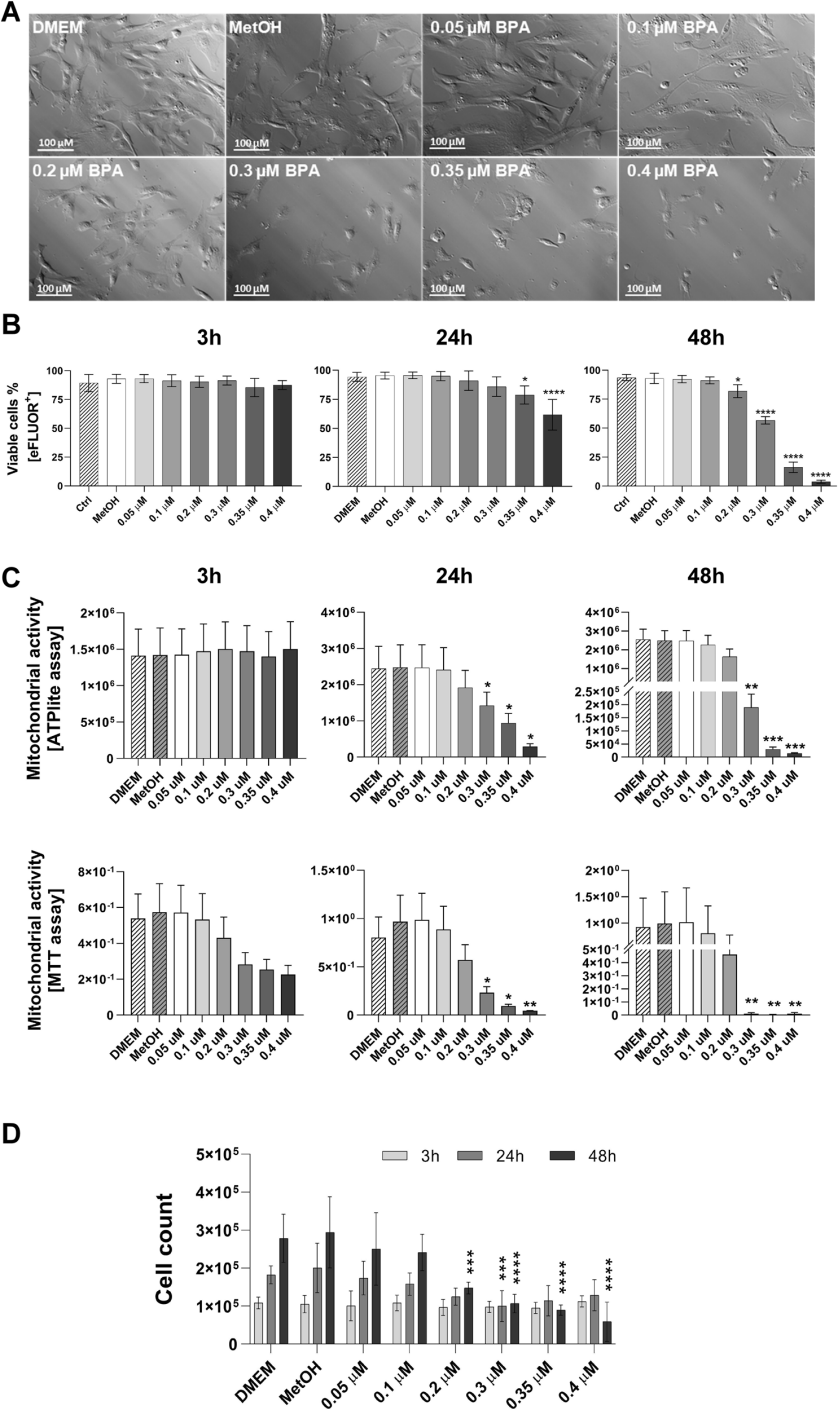
Assessment of hAMSC viability after 3, 24 and 48 h of exposure to increasing BPA concentrations. Morphological differences in hAMSC after 24 h of exposure with increasing concentrations of BPA (0.05, 0.1, 0.2, 0.3, 0.35, and 0.4 μM) (**A**). Images were acquired at ×20 magnification; scale bar, 100 μm. **B** Quantification of viable cells following 3, 24, and 48 h of BPA exposure, assessed via eFLUOR-positive staining. **C** Mitochondrial activity in hAMSCs after 3, 24, and 48 h of exposure, evaluated using MTT and ATPlite assays. **D** Absolute cell counts obtained by flow cytometry corroborate previously reported findings. Data are presented as mean ± SD from at least three independent experiments (*n* ≥ 3). Statistical significance was determined relative to the control condition (MetOH): **p < 0.01, ***p < 0.001, ****p < 0.0001.

We first investigated how increasing concentrations of BPA affect the viability of hAMSC. Methanol (MetOH), the solvent used to dissolve BPA, served as the experimental control and corresponded to the highest solvent concentration used to prepare the BPA. Following 24 h of exposure, morphological alterations were evident in hAMSCs exposed to increasing BPA concentrations (Fig. 1A). Cells treated with 0.05 and 0.1 μM BPA displayed a morphology comparable to that of MetOH-treated cells and those maintained under standard DMEM culture conditions.

On the other hand, cells exposed to higher concentrations (0.2, 0.3, 0.35, and 0.4 μM BPA) lost the characteristic spindle-like morphology of mesenchymal cells, transitioning to a rounded shape (Fig. 1A).

To further assess the impact of BPA on hAMSC viability, we quantified the percentage of eFluor-positive cells at 3, 24, and 48 h post exposure. No significant differences were observed at 3 h (Fig. 1B, left panel). However, a decline in cell viability was evident after 24 h, becoming statistically significant at BPA concentrations of 0.35 μM and higher in comparison to MetOH-treated cells (Fig. 1B, central panel). This effect was exacerbated at 48 h, with significant reductions observed at concentrations of 0.2 μM BPA and above (Fig. 1B, right panel).

Mitochondrial activity, a key indicator of cellular viability, was further evaluated using the MTT and ATPlite assays at all time points. Both assays revealed a pronounced reduction in cell viability, particularly in cells exposed to the highest BPA concentrations. A significant decrease was first detected at 0.3 μM BPA following 24 h of exposure and became more pronounced at 48 h (Fig. 1C). These findings were corroborated by absolute cell counts obtained via flow cytometry. As shown in Fig. 1D, a marked reduction in total cell number was observed after 24 h of exposure, reaching statistical significance at 0.3 μM BPA. This trend persisted at 48 h, with significant decreases occurring at concentrations of 0.2 μM and higher. No variations in cell count were observed at 3 h (Fig. 1D). All statistical analyses were conducted by comparing BPA-treated cells to the MetOH control condition.

### Increasing BPA concentrations induce oxidative stress in hAMSC initiating an antioxidant response

**Fig. 2:**
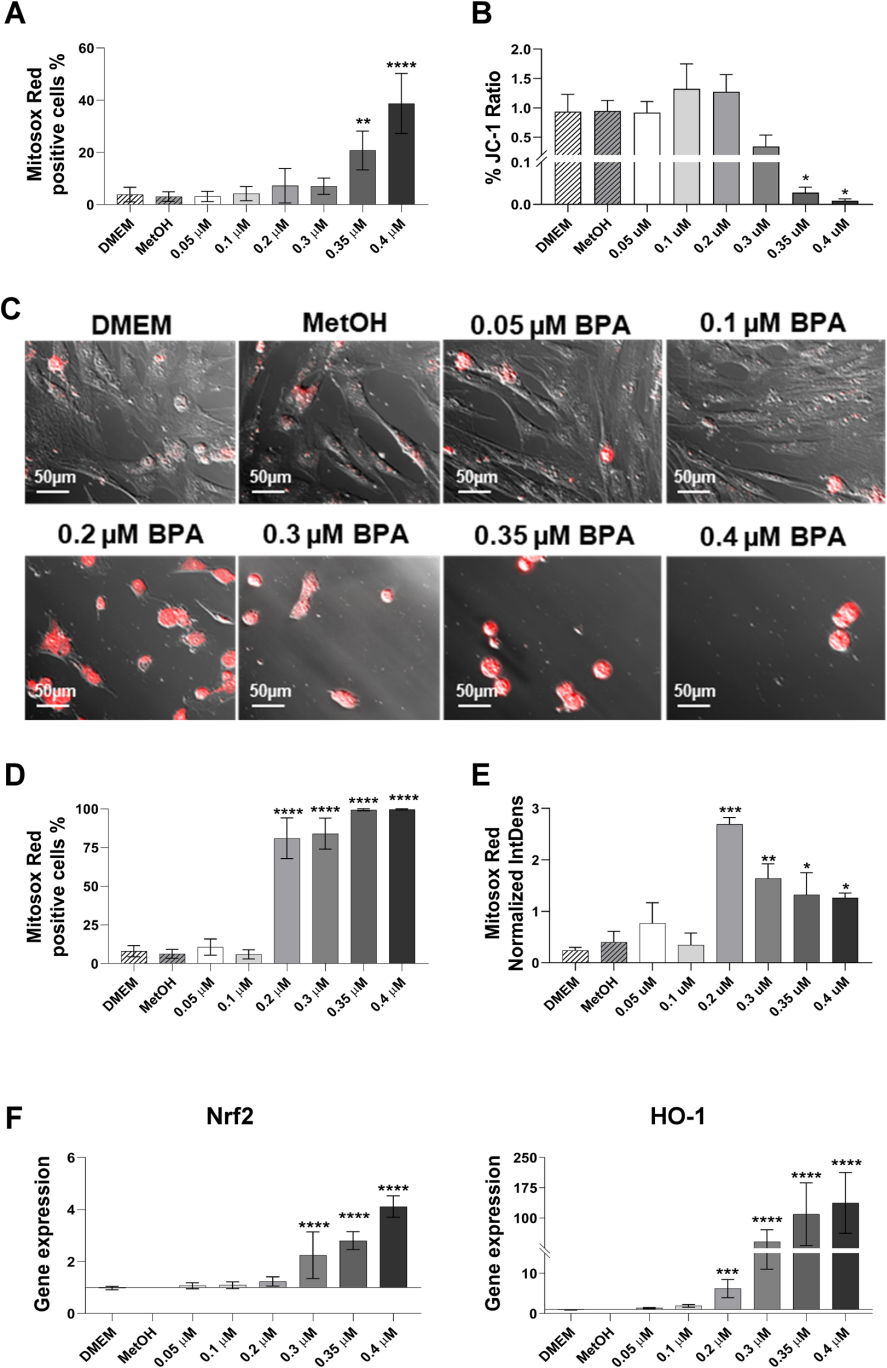
Evaluation of hAMSC oxidative stress after 24 h of exposure to increasing BPA concentrations. MtROS production in hAMSC was quantified in flow cytometry and in immunofluorescence using the MitoSOX Red fluorescent dye, after 3 and 24 h of exposure to increasing concentrations of BPA (0.05, 0.1, 0.2, 0.3, 0.35, and 0.4 μM). Results acquired in flow cytometry are presented as the percentage of MitoSOX Red-positive cells (**A**). Mitochondrial membrane potential (Δψm) variation was assessed using the JC-1 probe and representing the ratio, indicative of mitochondrial depolarization, following BPA exposure (**B**). Immunofluorescence images of hAMSC after 24 h of BPA exposure show MitoSOX Red-positive cells (red signal) overlaid on bright-field images (**C**), acquired at ×20 magnification (scale bar, 50 μm). The total number of MitoSOX Red-positive cells was quantified and is reported in (**D**). Fluorescence intensity of MitoSOX Red, measured as Normalized Integrated Density, is presented in (**E**). The antioxidant response of hAMSC was evaluated by quantifying Nrf2 and HO-1 gene expression after 24 h of BPA exposure, expressed as fold-change relative to the control condition (MetOH) (**F**). Histograms represent mean ± SD from *n* = 4 independent experiments. Statistical analysis was performed versus the control condition: *p* < 0.01 (*), *p* < 0.001 (**), *p* < 0.0001 (****).

To investigate the impact of BPA on mitochondrial oxidative stress, we assessed mtROS production at 3 (Supplementary Fig. 1) and 24 h (Fig. 2) post exposure. We excluded the 48-h time point due to excessive toxicity at the highest BPA concentrations. Flow cytometry analysis using MitoSOX Red revealed a concentration-dependent increase in mtROS-positive cells. At 3 h, this increase was modest but reached statistical significance at BPA concentrations ≥0.35 µM (Supplementary Fig. 1). After 24 h, mtROS levels were markedly elevated, with significant increases observed at concentrations ≥0.35 µM BPA (Fig. 2A, left panel).

Given the central role of mitochondrial function in oxidative stress generation [24], we next assessed mitochondrial membrane potential (Δψm) using JC-1 staining. At 24 h, cells exposed to 0.3 and 0.4 µM BPA exhibited a significant reduction in the JC-1 fluorescence ratio, indicative of mitochondrial depolarization (Fig. 2B). This decline closely mirrored the increase in mtROS levels and was consistent with findings from MTT and ATPlite assays (Fig. 1C), further supporting a BPA-induced impairment of mitochondrial function.

Immunofluorescence analysis corroborated these observations, showing a dose- and time-dependent increase in MitoSOX Red fluorescence, indicative of elevated mtROS levels (Fig. 2C). At 3 h, the number of MitoSOX Red-positive cells was significantly increased starting from 0.3 µM BPA (Supplementary Fig. 1), while at 24 h, this effect became more pronounced, with significant increases observed from 0.2 µM BPA (Fig. 2D). Control conditions (DMEM and methanol [MetOH]) showed no appreciable changes in mtROS levels, confirming the specificity of BPA-induced oxidative stress.

Fluorescence intensity quantification at the highest BPA concentration (0.4 µM) revealed a reduction in signal intensity at 24 h, in contrast with flow cytometry results. This discrepancy likely arises from methodological differences, as flow cytometry measures the total number of mtROS-positive cells, whereas fluorescence intensity in immunofluorescence can be affected by morphological alterations and variations in cell density within the field of view (Fig. 2E). Conversely, integrated density analysis at 3 h showed a significant increase in MitoSOX Red fluorescence intensity starting from 0.2 µM BPA, further supporting the early onset of oxidative stress (Supplementary Fig. 1).

In response to oxidative stress, hAMSC activated an antioxidant response involving the transcription factor Nrf2 and its downstream effector, HO-1 [25, 26]. At 3 h, Nrf2 expression showed a mild increase only at the highest BPA concentration (0.4 µM), while HO-1 expression was significantly upregulated (Supplementary Fig. 1). After 24 h, Nrf2 expression was significantly increased from 0.3 µM BPA onwards, accompanied by a robust upregulation of HO-1 expression at concentrations ≥0.35 µM BPA (Fig. 2F, right panels).

No significant differences were observed between the DMEM and MetOH control conditions; thus, MetOH, representing the highest solvent concentration used, was selected as the control for subsequent experiments.

### Increasing concentrations of BPA modulate the expression of inflammasome-related genes in hAMSC, without affecting IL-1β secretion

**Fig. 3:**
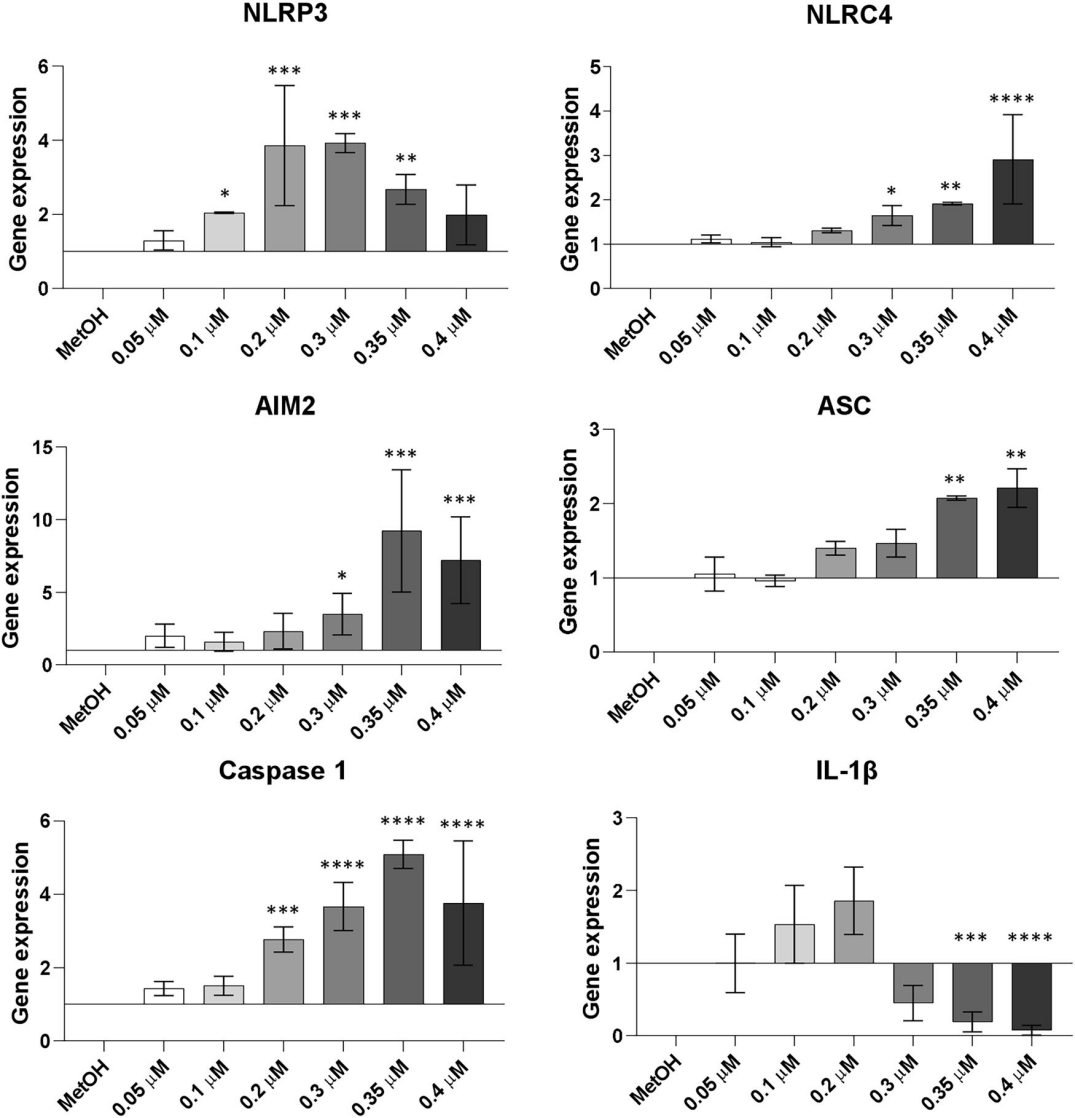
Evaluation of inflammasome recruitment and activation in hAMSC after 24 h of exposure to increasing BPA concentrations. Inflammasome formation and activation were assessed by examining the genetic expression of both structural and functional components of the complex (NLRP3, NLRC4, AIM2, ASC, caspase 1, IL-1β) (Fig. 3) and the secretion of effector protein (IL-1β) (Supplementary Table 1) after exposure to increasing BPA concentrations (0.05, 0.1, 0.2, 0.3, 0.35, and 0.4 μM) with or without ATP (1.5 mM). The figure illustrates alterations in gene expression, presented as fold-change relative to the control condition (MetOH). Histograms represent the mean values ± SD from *n* = 3 independent experiments. Statistical analysis was performed versus the control condition: *p* < 0.01(**), *p* < 0.001(***), *p* < 0.0001(****).

MtROS is a key activator of the inflammasome [27]. Considering the significant increase in mtROS production (Fig. 2A, B), we investigated the recruitment and the activation of the inflammasome complex in hAMSC exposed to increasing BPA concentrations. Particularly, we assessed the genetic expression of structural and effector components from 3 inflammasomes (NLRP3, NLRC4, AIM2) after 24 h of BPA exposure. Among these sensor proteins, which form multimeric complexes in response to stimuli in the cytoplasm, NLRP3 gene expression significantly increased with 0.2 and 0.3 μM BPA concentrations, returning to control levels at higher BPA concentrations (Fig. 3). Differently, genes encoding for NLRC4 and AIM2 sensor proteins, showed a significant upregulation following treatment with higher BPA concentrations (0.3; 0.35 and 0.4 μM). These results suggest varied effects of BPA on the diverse inflammasome complexes. Similar trends were observed for two other key players of the inflammasome, ASC and caspase 1, which exhibited significant upregulation with exposure to 0.35 μM and 0.2 μM BPA, respectively (Fig. 3). IL-1β, the final effector of the inflammasome cascade, showed a genetic expression pattern similar to NLRP3, with a significant increase compared to control after exposure to 0.2 μM BPA, and a significant decrease after exposure to 0.35 and 0.4 μM BPA (Fig. 3). However, unlike the genetic findings, IL-1β protein expression was undetected after exposure to increasing BPA concentrations, as assessed by ELISA (Supplementary Table 1). The lack of IL-1β production may indicate that BPA acts solely as a priming agent in inflammasome complex activation, where gene transcription occurs. Nevertheless, even after exposing the cells to a second stimulus adenosine triphosphate (ATP) to complete the activation of the complex [28, 29], IL-1β production was not observed (Supplementary Table 1).

### Increasing concentrations of BPA induce the gene expression of p53, p21 and p27 genes in hAMSC

**Fig. 4:**
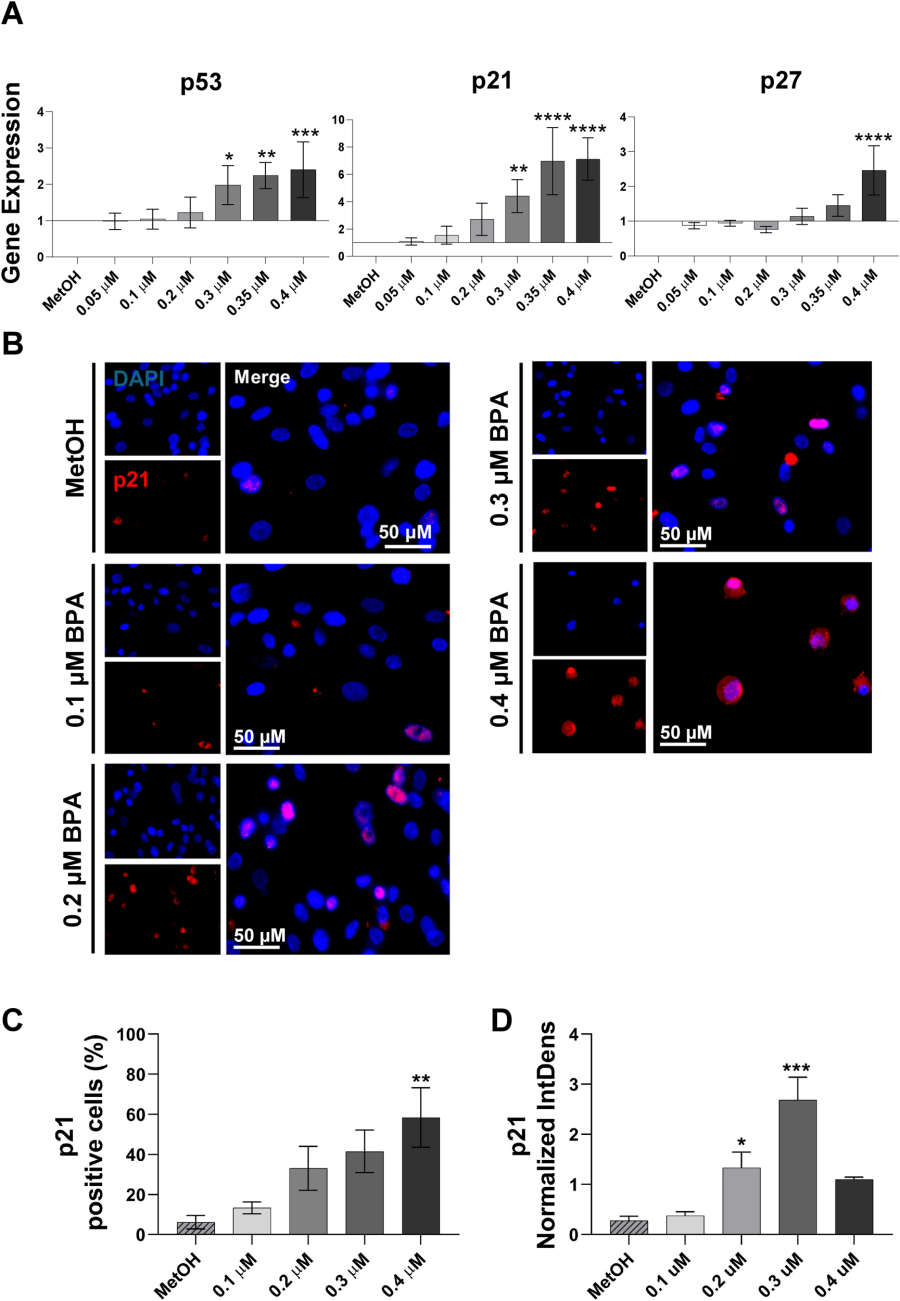
Investigation of cell cycle gene expression in hAMSC after 24 h of exposure to increasing BPA concentrations. Expression of p53, p21 and p27 cell-cycle regulating genes was analysed by RT-PCR 24 h after exposure to increasing BPA concentrations (0.05, 0.1, 0.2, 0.3, 0.35 and 0.4 μM). Results are presented as fold-change relative to control conditions (MetOH) (**A**). Furthermore, p21 protein expression and its nuclear translocation in hAMSC were assessed 24 h after exposure to increasing BPA concentrations (0.1, 0.2, 0.3, and 0.4 μM) using immunofluorescence analysis. Pictures were acquired at ×20 magnification (scale bar, 50 μm) (**B**). p21-positive cells were identified by a rosy-red signal, while nuclei were stained with DAPI (blue). The total number of p21 positive cells was quantified and is reported in (**C**). Fluorescence intensity of p21, measured as Normalized Integrated Density, is presented in (**D**). Histograms represent the mean values ± standard deviation from *n* = 4 (**A**) and *n* = 3 (**B**) independent experiments. Statistical analysis was performed versus the control condition represented by the MetOH: *p* < 0.01(**), *p* < 0.001(***), *p* < 0.0001(****).

In response to oxidative stress, cells can activate specific damage-response mechanisms, leading to cell cycle arrest, and potentially inducing senescence [30]. To investigate whether increased ROS production triggers these defensive mechanisms in hAMSC, we examined the expression of p53 gene, a primary transcription factor recruited under stressful conditions. Results from RT-PCR revealed a significant upregulation in p53-encoding gene, starting from 24 h of exposure with 0.3 μM BPA (Fig. 4A, left panel). Furthermore, we investigated changes in the expression profiles of p21 and p27, two master transcriptional factors responsible for cell cycle arrest and senescence induction. Indeed, the recruitment of these two cycline-dependent kinase (CDK) inhibitors is often mediated by p53 expression [31]. As depicted in Fig. 4A (central and right panels), significant upregulation of p21 and p27 genes was observed, with significant values reached from 0.3 μM BPA concentration for p21, and only at the highest tested concentration for p27. To determine whether the increased p21 gene expression correlated with functional activity, we assessed its nuclear translocation by immunofluorescence analysis after exposure to increasing BPA concentrations (0.1, 0.2, 0.3, 0.4 μM). As observed in Fig. 4B, increased nuclear translocation of *p21* was evident even at the lowest concentration tested, with significance reached only at the highest tested concentration of 0.4 μM BPA (Fig. 4C). To further investigate the impact of BPA exposure on *p21* protein levels, we quantified fluorescence intensity as a surrogate measure of protein accumulation. As shown in Fig. 4D, fluorescence intensity analysis revealed a dose-dependent increase in *p21* levels, supporting the hypothesis that BPA-induced *p21* upregulation translates into increased protein expression.

### Increasing concentrations of BPA induce senescence in hAMSC with the acquisition of the SASP profile

**Fig. 5:**
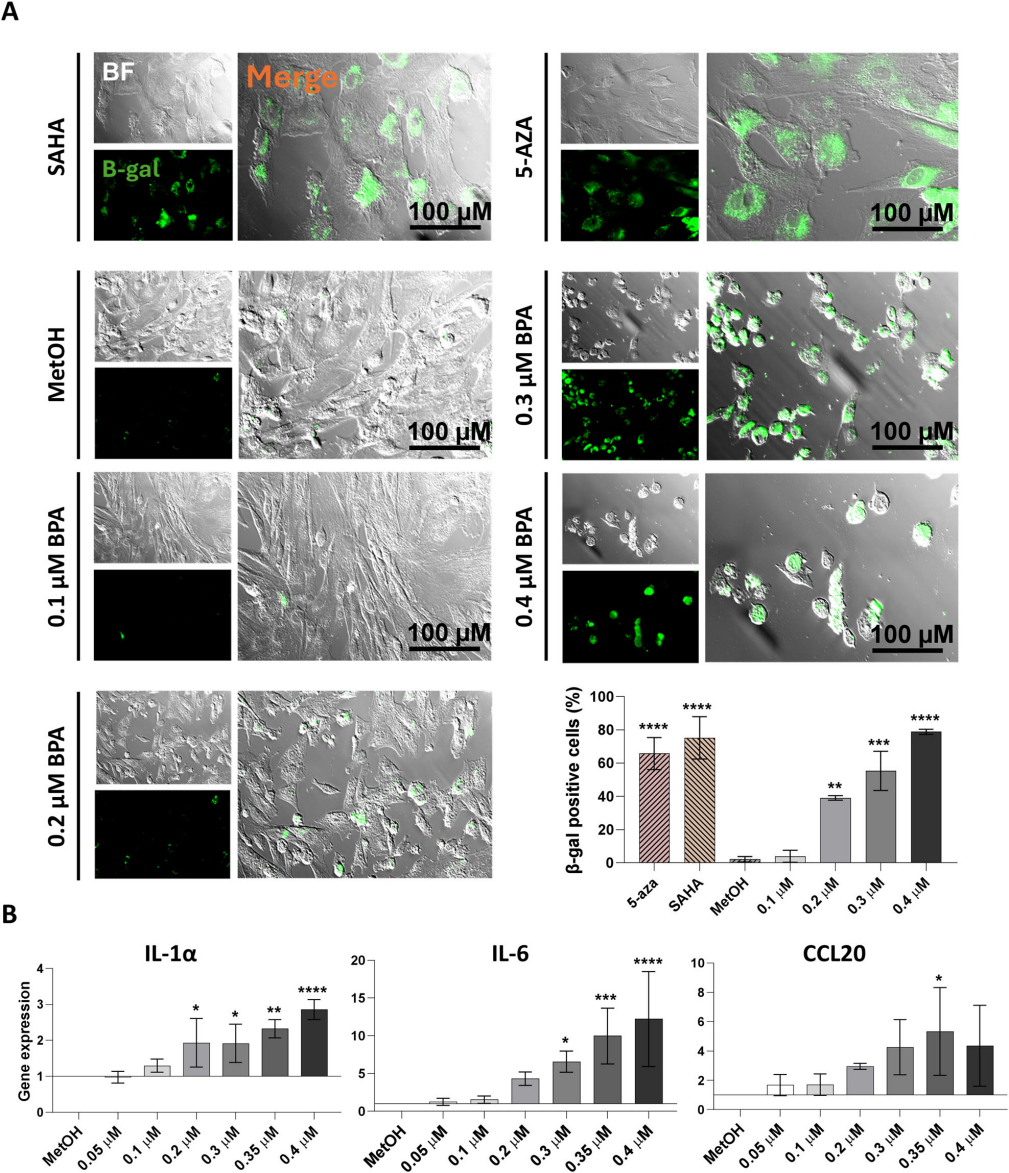
Investigation of senescent induction in hAMSC after 24 h of exposure to increasing BPA concentrations. β-galactosidase (β-gal) activity in hAMSC was evaluated by immunofluorescence analysis 24 h after exposure to increasing concentrations of BPA (0.1, 0.2, 0.3, and 0.4 μM) (**A**). β-gal-positive cells were identified by a green signal. Pictures were acquired at ×20 magnification (scale bar corresponds to 100 μM in **A**). The percentage of senescent cells ((β-gal) positive) detected by immunofluorescence analysis was represented. Expression of senescent-associated secretory phenotype (SASP) molecules (IL-6, IL-1α and CCL20) were analysed by RT-PCR 24 h after BPA exposure and presented as fold-change relative to the control (MetOH) (**B**). Results are represented as histograms showing mean values ± SD from *n* = 3 independent experiments. Statistical analysis was performed versus the control condition: *p* < 0.01(**), *p* < 0.001(***), *p* < 0.0001(****).

To validate our findings and confirm the acquisition of a senescent phenotype in hAMSC, we assessed β-galactosidase activity in cells treated with increasing BPA concentrations. Results were compared with those obtained from hAMSC treated with the epigenetic senescence inducers 5-aza-2’-deoxycytidine (5-aza) and SAHA, serving as positive controls [32]. β-galactosidase (β-gal)-positive cells exhibited green perinuclear staining, with a higher frequency and intensity in cells exposed to 0.3 and 0.4 μM BPA concentrations compared to the negative control (MetOH-treated cells), and similar to the positive controls. As shown in the representative histograms (Fig. 5A), the percentage of senescent cells reached significance starting from 0.2 μM BPA concentration and exceeded 70% positivity for hAMSC exposed to 0.4 μM BPA concentration (as observed in 5-aza and SAHA-treated cells).

Since the senescence state is characterized by the secretion of bioactive factors, collectively known as senescence-associated secretory phenotype (SASP) [33], we further analysed the expression of IL-6, IL-1α, and CCL20. Similarly, to the acquisition of β-gal positivity (Fig. 5A), a significant upregulation in the genetic expression was observed starting from the concentration of 0.2 μM BPA for IL-1α (Fig. 5B, left panel), 0.3 μM for IL-6 (Fig. 5B, central panel), and 0.35 μM for the chemokine CCL20 (Fig. 5B, right panel).

### Toxicity induced by increasing concentrations of BPA culminates in the induction of apoptosis with dysregulated expression of Bax and Bcl-2 genes in hAMSC

**Fig. 6:**
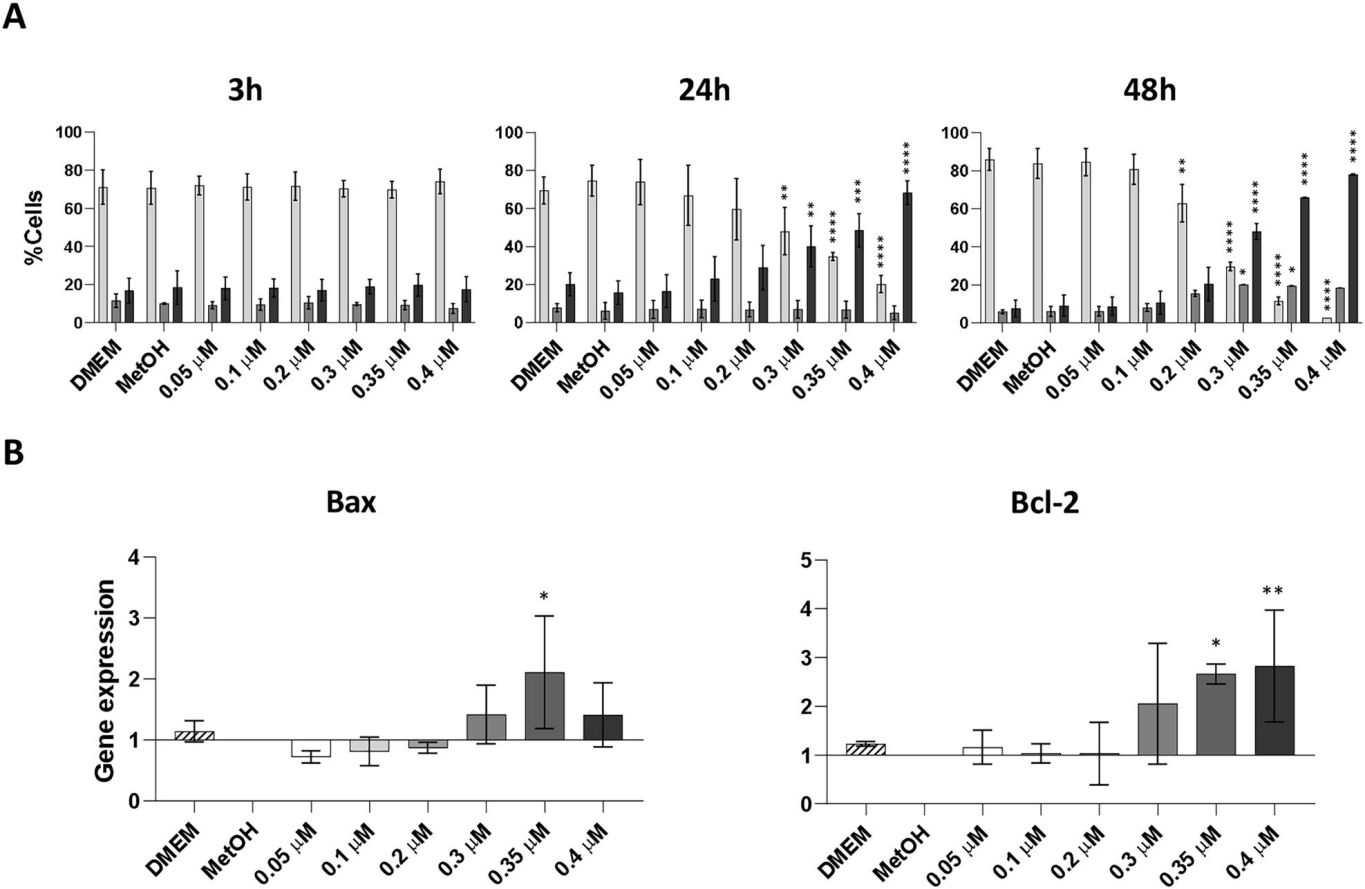
Investigation of apoptosis induction in hAMSC after 3, 24 and 48 h of exposure to increasing BPA concentrations. Percentage of live, early and late apoptotic cells was measured by Annexin-PI fluorogenic dyes 3, 24 and 48 h after exposure to increasing concentrations of BPA (0.05, 0.1, 0.2, 0.3, 0.35, and 0.4 μM) (**A**). Expression of pro-apoptotic (Bax) and anti-apoptotic (Bcl-2) genes was analysed by RT-PCR 24 h after BPA exposure and presented as fold-change relative to the control condition (MetOH) (**B**). Results are represented as histograms showing mean values ± SD from *n* = 3 independent experiments. Statistical analysis was performed versus the control condition: *p* < 0.01(**), *p* < 0.001(***), *p* < 0.0001(****).

Oxidative stress can lead to cell death when all activated processes are insufficient to repair the damage [34]. In this regard, we assessed the extent of cells undergoing activation of the apoptotic pathway after exposure to increasing concentrations of BPA, using the PI-Annexin V assay. Analyses conducted 3, 24, and 48 h after exposure to BPA revealed no variations at 3 h (Fig. 6A, left panel). However, 24 h after BPA exposure, the percentage of live cells began to decline, reaching significance starting from exposure with 0.3 μM BPA concentration. Concurrently, the number of late apoptotic cells significantly increased at the same BPA concentration (Fig. 6A, central panel). This effect intensified at 48 h, with a significant reduction in the percentage of live cells observed from 0.2 μM BPA and a significant increase in late apoptotic cells from 0.3 μM BPA (Fig. 6A, right panel). No changes in the percentage of early apoptotic cells were detected 3, 24, and 48 h after BPA treatment.

**Fig. 7:**
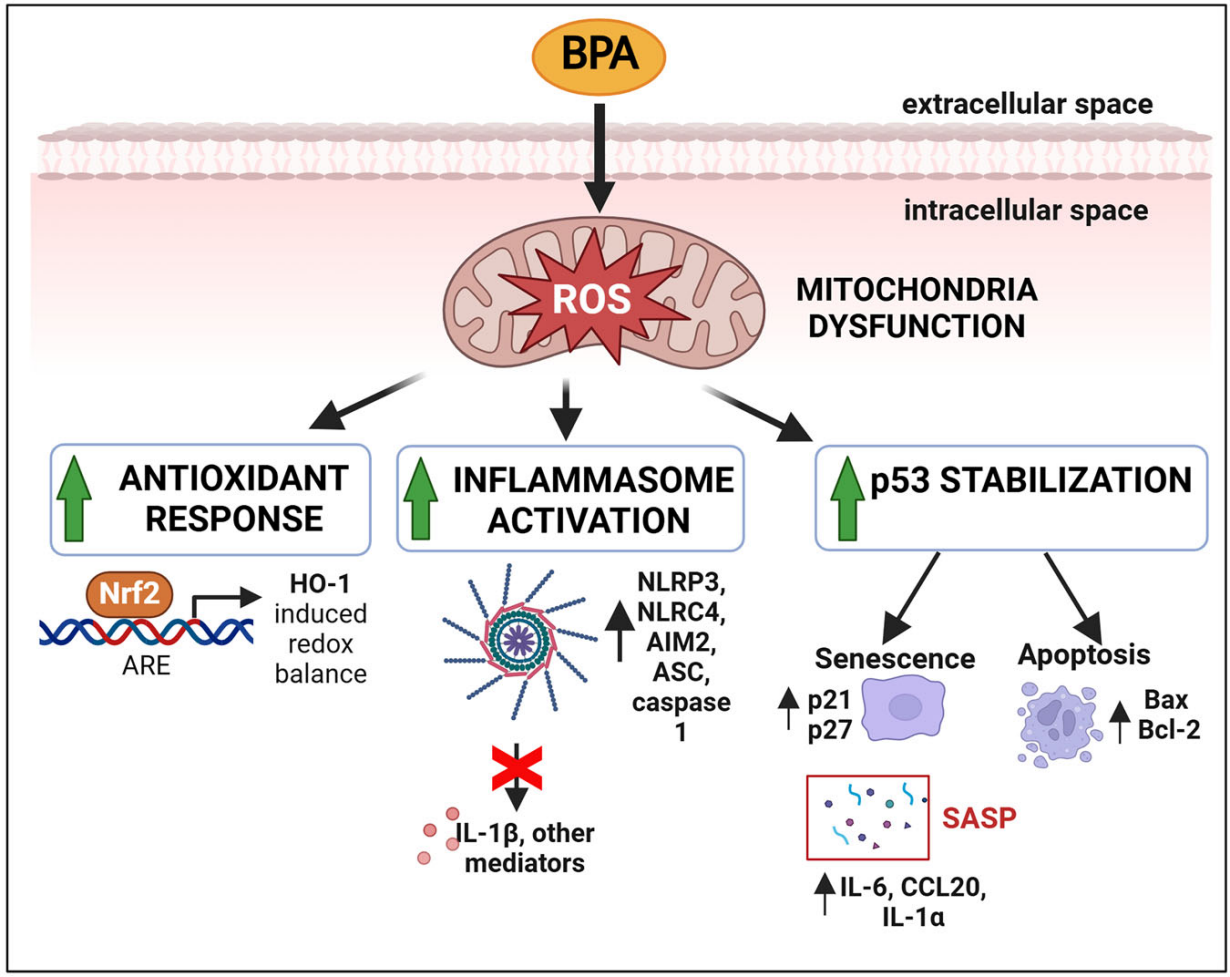
Proposed outline of the signalling pathways activated in hAMSC after exposure to increasing BPA concentrations. Alteration in mitochondrial function, evidenced by enhanced production of ROS in hAMSC after BPA exposure, trigger the downstream activation of several signalling pathways. ROS accumulation activates an antioxidant response, which is marked by an elevated production of Nrf2 and HO-1. Concurrently, the high ROS level induce sterile inflammation, resulting in increased transcription of factors involved in inflammasome complex formation and activation. However, this increased transcription does not translate to higher production of IL-1β, the downstream effector of the inflammasome pathway. Instead, oxidative stress promotes p53 stabilization and upregulates p21 and p27 genes, as well as components of the senescence-associated secretory phenotype (SASP). Ultimately, the senescent state serves as a prelude to apoptosis, which occurs when hAMSC are exposed to the highest BPA concentrations.

Additionally, the genetic expression of the two well-known pro-apoptotic and anti-apoptotic genes, Bax and Bcl-2, was dysregulated after 24 h of BPA exposure, with significant values reached with 0.35 and 0.4 μM BPA concentrations (Fig. 6B). A summary diagram illustrating the impact of BPA on hAMSC is shown in Fig. 7.

## Discussion

In recent years, the threat of microplastics to human health has been widely discussed, particularly concerning pregnant women and infants, who are especially sensitive to toxicants exposure [5]. A landmark discovery in this topic was published in 2021 by Ragusa and colleagues, which demonstrates the presence of microplastics in different areas of the human term placenta (the fetal portion, the maternal portion, and the amniotic membrane) [11]. Considering the crucial role of the placenta as the interface between the fetus and the external environment, the discovery of MPs within it raises concerns about the potential negative effects of microplastic pollution on pregnancy progression. In particular, MPs toxicity is mostly attributed to plasticizers [35], among which BPA has been extensively studied for its possible link with obstetric complications, such as preeclampsia, fetal growth restriction, miscarriage, and preterm birth [17]. The mechanisms by which BPA influences these diseases are still unclear, prompting numerous studies to explore its intracellular actions.

Our research is the first to examine the effects of BPA on hAMSC. The aim is not only to assess BPA’s impact on these cells but also to uncover the pathways through which it operates.

Exposure to BPA significantly compromised hAMSC viability in a dose- and time-dependent manner. A pronounced reduction was evident following 24 h of exposure to the highest BPA concentrations tested (0.35 and 0.4 μM), consistent with previous reports of BPA-induced cytotoxicity. Notably, mitochondrial activity, a key indicator of cell viability, was significantly reduced at 0.3 μM BPA after 24 h, as demonstrated by MTT and ATPlite assays, with further declines observed at 48 h. Flow cytometry-based absolute cell counts corroborated these findings, revealing a significant reduction in total cell number at 0.3 μM BPA after 24 h, which became more pronounced at 48 h at concentrations of 0.2 μM and higher. No significant changes were detected at the 3-h time point, reinforcing the notion that BPA-induced cytotoxic effects emerge progressively over time. Similar viability declines have been observed in RAW 264.7 macrophages and MLO-Y4 osteocytes exposed to 50 and 200 μM BPA, respectively, for 24 h [36]. Similarly, 10 µM BPA induced cytotoxicity and reduced viability in ovarian cancer cell lines after 48 h of exposure [37], while 100 mM BPA negatively affected BeWo trophoblast viability after 24 h of exposure [38]. Although these concentrations exceed those used in our experiments, they confirm BPA’s capacity to decrease cellular viability. It noteworthy that these studies employ cell lines, which may be more resistant than primary cells like hAMSC. Notably, research on human fetal lung fibroblasts showed that BPA affected the expression of genes involved in cell cycle regulation at concentrations comparable to those used in our study [39].

Furthermore, the BPA concentrations employed in our experiments were selected to reflect exposure levels relevant to human gestation. This selection was based on recent studies assessing serum and urine BPA concentrations in pregnant women to evaluate possible association with gestational complications [12, 13]. We selected six different doses, including at least one with negligible effects (0.05 µM) and one causing a significant reduction (0.4 µM) on cell viability, comparable to the concentrations found in the blood and urine of woman with complicated pregnancies [12, 13]. Furthermore, our range aligns with the lowest observable adverse effect level (LOAEL) defined for the in vitro evaluation of BPA toxicity, which corresponds to 2.19 × 10^−7^ M [40, 41]. In this study, we define concentrations closer to this value, ranging from nanomolar to millimolar, as low doses. BPA toxicity has been extensively linked to oxidative stress, with evidence reported across in vitro [42], in vivo [43], and human studies [44, 45].

Our findings support these observations, identifying mitochondrial dysfunction and oxidative stress as key mediators of BPA-induced cytotoxicity in hAMSC. Notably, MitoSOX Red staining revealed a significant accumulation of mtROS as early as 3 h post exposure, persisting at 24 h in cells treated with BPA concentrations ≥0.35 µM. This increase was accompanied by a dose- and time-dependent loss of mitochondrial membrane potential (Δψm), as demonstrated by JC-1 staining, suggesting that BPA-induced mitochondrial dysfunction drives oxidative stress accumulation. A decline in Δψm disrupts the electron transport chain (ETC), a key site of ATP production, leading to electron leakage at complexes I and III and the subsequent formation of superoxide radicals (O₂⁻•) [46]. In another article it has been reported that BPA exposure induced cytotoxicity in hBMSCs in a dose- and time-dependent manner, accompanied by increased lipid peroxidation. Notably, cell death was significantly mitigated by superoxide dismutase mimetics (MnTBAP and MnTMPyP), but not by catalase, glutathione, or inhibitors of NOS and xanthine oxidase, highlighting superoxide anions as key mediators of BPA toxicity. Furthermore, BPA exposure led to a decline in nuclear β-catenin and cyclin D1 levels, which was reversed by MnTBAP treatment. Inhibition of GSK3β via LiCl₂ also restored nuclear β-catenin and attenuated cytotoxicity, suggesting that BPA disrupts β-catenin signaling through superoxide anion overload [47].

These results align with previous studies reporting BPA-induced ETC impairment and oxidative stress in various cell types. Indeed it has been reported that HepG2 hepatoma cells exposed to nanomolar BPA concentrations showed increased ROS production after 24 h [48] and reduced mitochondrial membrane potential after 6 h [49]. GT1-7 hypothalamic neurons exposed to micromolar BPA concentrations exhibited increased mtROS production after 6 h [50], while bovine granulosa cells showed increased oxidative stress and upregulation of antioxidant enzymes when exposed to low BPA concentrations for 12 h [42].

Beyond ETC disruption, BPA’s lipophilic nature may facilitate its incorporation into mitochondrial membranes, directly interfering with ETC protein function and exacerbating ROS generation. Additionally, BPA has been reported to dysregulate intracellular calcium homeostasis through interactions with estrogen receptors and the induction of endoplasmic reticulum (ER) stress [51]. Excessive mitochondrial calcium uptake can trigger mitochondrial permeability transition pore (mPTP) [52] opening, leading to Δψm destabilization and further amplifying ROS production. These processes likely contribute to the self-perpetuating oxidative damage cycle observed in our study, in which mtROS-induced lipid, protein, and DNA oxidation progressively impair mitochondrial function over time. In response to oxidative stress, hAMSC activated an antioxidant defense mechanism involving the transcription factor Nrf2 and its downstream effector heme oxygenase-1 (HO-1) [53]. While Nrf2 activation is a key adaptive response aimed at mitigating oxidative damage, our findings suggest that it is insufficient to restore mitochondrial homeostasis at higher BPA concentrations. This is evident from the sustained loss of Δψm, excessive mtROS accumulation, and progressive cell death observed at 24 and 48 h. Dysregulated Nrf2 signaling has been implicated in BPA toxicity in both in vitro and in vivo models, with studies reporting altered antioxidant enzyme expression and oxidative damage across multiple tissues. In line with these findings, we observed a significant upregulation of Nrf2 and its downstream effector, heme oxygenase-1 (HO-1), 24 h after BPA exposure, suggesting an adaptive cellular response to oxidative stress. Nrf2 plays a critical role in cellular detoxification mechanisms, and its involvement in BPA-mediated toxicity is well-documented. In human B cells, exposure to 100 µM BPA induced autophagy via Nrf2-mediated upregulation of pro-survival factors Atg7 and Beclin1 [54], while concentrations of 50 and 100 µM BPA led to increased Nrf2 expression [55]. In vivo, low-dose BPA exposure in frog embryos resulted in teratogenesis, DNA damage, and dysregulated expression of antioxidant enzymes (SOD1, NQO1, and CAT), likely through disrupted Nrf2 signaling [56], given its essential role in human embryonic stem cell differentiation [57] and stem cell property maintenance [58]. Thus, Nrf2 dysregulation may be a cellular response to mitigate BPA toxicity but could have deleterious effects on placental-resident stem cells during gestation

Dysregulated Nrf2 signaling has been implicated in BPA toxicity in both in vitro and in vivo models, with studies reporting altered antioxidant enzyme expression and oxidative damage across multiple tissues [49–54].

As previously mentioned, heightened oxidative stress activates numerous signaling pathways, including antioxidant response, inflammation, apoptosis, and senescence [59]. BPA-induced oxidative stress has been linked to inflammation, apoptosis, and mitochondrial dysfunction in the colon and liver of mice [60]. Picomolar concentrations of BPA disrupt proliferative and inflammatory pathways in human endothelial cells through oxidative stress [61]. Considering that ROS production can activate the inflammasome complex [27], we investigated whether BPA exposure induces the activation of a well-characterized inflammasome, the nucleotide-binding oligomerization domain (NOD)-like receptor containing pyrin domain 3 (NLRP3), in hAMSC. We observed upregulation of NLRP3, caspase 1, ASC, and IL-1β genes, but not the protein expression of the final effector IL-1β in hAMSC supernatant. This result suggests a blockade in the activation of the inflammasome complex. However, the complete inflammasome activation requires an initial priming stimulus that induces the expression of inflammasome-related genes, followed by a second stimulus to trigger the release of the final effectors [28]. In our experiments, even the addition of a second stimulus (ATP) failed to induce IL-1β production and NLRP3 inflammasome activation.

Solely two other studies have documented the involvement of NLRP3 inflammasome, in osteocytes exposed to 100 and 200 µM BPA [36] and in RAW 264.7 murine macrophages exposed to micromolar BPA concentrations [62]. They show contrasting results: the former observed increased expression of inflammasome-associated proteins and induction of pyroptosis, the latter reported inactivation of the NLRP3 inflammasome and lack of IL-1β and IL-18 production. These observations highlight a different effect of BPA on this specific intracellular pathway, which nonetheless result in dysregulated cellular response in both cases.

Based on our results, our hypothesis was that BPA-mediated toxicity may be severe enough to elicit a distinct cellular response.

Following exposure to the highest BPA concentrations (0.3, 0.35, and 0.4 µM), we observed upregulation of p53 gene, along with p21 and p27 cell-cycle regulators, markers typically associated with cellular senescence. p53, which plays a crucial role in determining cell fate under stress condition, can induce p21 expression, leading to senescence or apoptosis. Senescence is characterized by irreversible cell-cycle arrest and secretion of inflammatory molecules, which trigger a senescent phenotype in neighboring cells as well [33].

Immunofluorescence analysis revealed nuclear localization of p21 starting at 0.2 µM BPA, coinciding with the onset of β-galactosidase activity in hAMSC. Additionally, BPA-exposed cells exhibited elevated levels of IL-1α, IL-6, and CCL20, key components of the senescence-associated secretory phenotype (SASP) [63], indicating that increasing BPA concentrations induce cellular senescence. These findings align with previous reports in both primary and prostate cancer cells, where BPA exposure (10–100 µM) upregulated p21 and p27, leading to cell cycle arrest via the EGFR/ERK/p53 signaling pathway [64]. Similarly, murine aortic endothelial cells treated with 100 nM to 5 µM BPA displayed increased β-galactosidase activity and upregulation of p16 and p21, hallmark markers of senescence [65]. Notably, the molecular mechanisms underlying BPA-induced senescence appear to be cell-type dependent. In C2C12 murine myoblasts, senescence occurred independently of p53, instead involving the p15-Rb1 pathway [66]. These findings underscore oxidative stress as a key driver of BPA-induced senescence while highlighting mechanistic differences across cell types.

In vivo experiments reported that low doses of BPA induce renal injury in adult rats, evidenced by histological alterations and increased cellular senescence [67].

In the context of BPA exposure, the induction of senescence state in hAMSC can be considered an adaptive stress response. Moreover, it is important to highlight that senescence is a required process during embryological development, but excessive senescence can be associated with negative pregnancy outcomes [68].

hAMSC may respond to BPA-induced oxidative stress by arresting cell cycle progression and proliferation, as evidenced by the dramatic decrease in cell numbers at higher BPA concentrations.

As suggested by the PI/Annexin evaluation, BPA-mediated toxicity can activate apoptosis starting from 24 h of exposure.

At higher BPA concentrations, a subset of hAMSC underwent apoptosis, as indicated by the upregulation of both pro-apoptotic *BAX* and anti-apoptotic *BCL-2*. Annexin V/PI staining confirmed apoptosis as the predominant mode of cell death, while necrosis remained negligible. The concurrent upregulation of *BAX* and *BCL-2* may initially appear contradictory; however, this paradox reflects the complexity of cellular stress responses, particularly under endocrine-disrupting conditions.

BPA’s estrogenic activity has been shown to modulate apoptotic pathways in a dose- and cell-type-dependent manner [69]. In murine ovarian granulosa cells, low-dose BPA exposure induced apoptosis via *BAX* upregulation and *BCL-2* downregulation, highlighting the intricate balance between pro- and anti-apoptotic signaling [70]. Additionally, estrogen itself has been shown to simultaneously upregulate *BAX* and *BCL-2*, as demonstrated in macrophages where 17β-estradiol (E2) increased *BCL-2* expression while promoting *BAX* translocation to mitochondria in an estrogen receptor-dependent manner [71].

These findings underscore the dynamic nature of BPA-induced cellular responses, in which mitochondrial dysfunction serves as a central hub linking oxidative stress, apoptosis, and senescence. The disruption of mitochondrial homeostasis and the oxidative stress-induced feedback loop likely underpin many of BPA’s toxic effects on placental cells.

## Conclusion

In conclusion, our results, consistent with current research, suggest that BPA is an environmental pollutant extremely toxic to cells, and specifically to hAMSC. Our findings go beyond current research by demonstrating, for the first time, the in vitro impact of environmentally relevant concentrations of BPA on hAMSC, elucidating pathways through which it acts. We observed a reduction in hAMSC viability and increased production of mtROS, associated with mitochondrial dysfunction. The increased oxidative stress appears to play a central role, triggering a series of downstream signaling pathways aimed to restore cellular homeostasis. Additionally, our in vitro evaluations suggest that hAMSC exposed to increasing BPA concentrations, partially arrest cell cycle progression, entering senescence, and partly undergo apoptosis, when toxicity is unsustainable. These results contribute to a broader understanding of BPA’s direct impact on placenta cells.

Considering the limitations inherent in investigations during pregnancy, these findings are significant for informing large-scale decisions, such as restricting BPA in certain products to safeguard the health of both the mother and her baby, as well as public health overall.

## Materials and methods

### Isolation and culture of hAMSC

Placentae were processed immediately after collection and hAMSC were isolated as previously described [72]. Freshly isolated hAMSC were expanded until passage 1 (p1) and then frozen for future experiments. Expansion was performed by plating cells at a density of 10^4^ cells/cm^2^ in Chang D medium (Irvine Scientific, Santa Ana, CA, USA) supplemented with 2 mM L-glutamine and and 1% P/S, at 37 °C in a 5% CO_2_ incubator. Cells with >98% expression of mesenchymal markers CD13 and CD90, <2% expression of hematopoietic marker CD45, and <2% expression of epithelial marker CD324 were utilized in this study.

### Treatment of hAMSC with BPA

To assess the effects of BPA, hAMSC p1 were thawed and seeded at a density of 40 × 10^4^ cells/cm^2^ in Chang D medium supplemented with 2 mM L-glutamine and 1% P/S and maintained in a standard cell culture incubator at 37 °C with 5% CO_2_. After cells adhesion to the plastic support, they were exposed to increasing concentrations of BPA for 3, 24 and 48 h. BPA powder (Sigma-Aldrich #239658) was dissolved in 90% methanol to create an intermediate solution (0.2 mM), which was then diluted in DMEM High Glucose medium (Euroclone, Milan, Italy) supplemented with 20% heat-inactivated FBS, 2 mM L-glutamine, and 1% P/S. hAMSC were exposed to six final concentrations of BPA: 0.05, 0.1, 0.2, 0.3, 0.35, and 0.4 μM BPA. Additionally, hAMSC were exposed to treatment with methanol (used to dissolve BPA) at the highest concentration present in the condition with 0.4 μM BPA. The range of doses and the exposure time were selected based on previous literature investigations to ensure that cells were exposed to environmentally relevant concentrations [40]. This selection was extensively addressed in the discussion.

### Evaluation of hAMSC mitochondrial functionality

Mitochondrial activity of hAMSC was assessed using the Thiazolyl Blue Tetrazolium Bromide (MTT) colorimetric assay (Sigma-Aldrich, Burlington, Massachusetts, USA) and the Cell Titer-Glo Luminescent Cell Viability assay (Promega, Madison, USA, #G7570), following the manufacturer’s protocols. Briefly, cells were plated and treated with BPA as previously described. After 24 h of exposure, mitochondrial functionality was assessed concurrently using the MTT assay and the Cell Titer-Glo assay. For the MTT assay, cells were incubated for 3 h with 0.5 mg/mL MTT, followed by overnight dissolution of intracellular MTT-formazan crystals in MTT-lysis solution (50% dimethylformamide in deionized H2O supplemented with 20 g SDS). Absorbance was measured at 550 nm using a VictorTM X4 plate reader. ATP production was determined using the Cell Titer-Glo kit, where cells were incubated for 20 min with 20 μL of Cell Titer-Glo reagent, then transferred in white support plates, and luminescence was measured using a VictorTM X4 plate reader.

### Evaluation of hAMSC viability, total count and apoptotic rate

To evaluate the impact of BPA on hAMSC viability, cells were harvested 3, 24, and 48 h after exposure to increasing concentrations of BPA. Subsequently, cells were stained with the eBioscienceTM Fixable Viability Dye eFluorTM 780 (Thermo Fisher Scientific, Waltham, Massachusetts, USA, # 65-0865-14), following the manufacturer’s instructions, to analyze viable (eFluor-negative) and dead (eFluor-positive) cells. Samples were acquired using FACS Symphony A3 BD, and the data were analyzed using FlowJo 10.8 software. The absolute cell count was determined by resuspending all samples in the same volume and acquiring at the same speed for a fixed time within the live cell gate.

Furthermore, to investigate the pro-apoptotic effect of BPA, we analyzed the positivity of hAMSC for FITC-Annexin V-propidium iodide (PI) kit (BD Biosciences, Franklin Lakes, New Jersey, USA, # 556547) according to the manufacturer’s instructions. After a 15-min incubation in darkness at room temperature, the samples underwent two washing steps with binding buffer. Samples were acquired using FACS Symphony A3 BD within an hour of staining. Collected data were analyzed using FlowJo version 10.8 software. Cells were categorized into the early apoptotic phase (Annexin V+/PI−), late apoptotic phase (Annexin V+/PI+), and necrotic phase (Annexin V-/PI+).

### Measurement of hAMSC oxidative stress

To assess oxidative stress in hAMSC, cells were thawed and seeded in Chang D medium supplemented with 2 mM L-glutamine and 1% P/S either at a density of 40 × 10^4^ cells/cm^2^ for flow cytometry evaluation or seeded at a density of 25 × 10^4^ cells/cm^2^ in IBIDI chambers (IBIDI, Gräfelfing, Germany, #81816) for immunofluorescence. ROS were detected using the MitoSOX Red fluorogenic dye (Life Technologies #M36008), following the manufacturer’s instructions. Briefly, 3 and 24 h after BPA exposure, hAMSC were incubated with MitoSOX Red (0.7 µM) for 20 min at 37 °C in the dark. Following incubation, cells were washed twice with PBS and acquired using FACS Symphony A3, and subsequently analysed using FlowJo 10.8 software, or using the MICA platform from Leica. For the acquisition via the MICA platform, we analysed a minimum of 200 cells across three fields for most conditions. However, due to a significant reduction of countable cells at the highest BPA concentrations, we increased the number of fields to achieve a sufficient cell count. In addition to cell counting, fluorescence intensity variation following BPA treatment was assessed using ImageJ software. For each image, the region of interest (ROI) was selected based on the cell area, and the mean fluorescence intensity was measured. Background fluorescence was subtracted from each measurement, and the data were reported as normalized integrated density. This quantification allowed for a standardized comparison of MitoSOX expression levels across different experimental conditions.

### JC-1 staining

hAMSCs were thawed and seeded in Chang D medium supplemented with 2 mM L-glutamine and 1% penicillin/streptomycin at a density of 40 × 10^4^ cells/cm^2^. After 24 h, cells were exposed to BPA at six final concentrations (0.05, 0.1, 0.2, 0.3, 0.35, and 0.4 μM) for 3 or 24 h. Cells were then washed twice with PBS and stained with JC-1 (Thermo Fisher Scientific #T3168) following the manufacturer’s protocol. Fluorescence was acquired using a FACS Symphony A3 (BD Biosciences) and analyzed with FlowJo 10.8 (BD Biosciences). JC-1 fluorescence was expressed as the JC-1 ratio, and data were plotted using GraphPad Prism.

### Gene expression analysis of hAMSC

For gene expression analysis, hAMSC were collected in RLT buffer (Qiagen, Frederick, MD, USA, # 79216) after 3 and 24 h of exposure to increasing concentrations of BPA. Samples were preserved at −80 °C until use. Upon thawing, total RNA extraction was performed using the EZ1 RNA Cell Mini Kit (Qiagen, Frederick, MD, USA # 959034) in a BioRobot EZ1 Advanced XL Workstation. Subsequently, cDNA synthesis was carried out using the iScript Advanced cDNA Synthesis Kit for RT-qPCR (Biorad, Hercules, California, USA, # 1725038 BUN). Then, the cDNA was pre-amplified with SsoAdvanced PreAmp Supermix (Biorad, Hercules, California, USA, #1725160).

Real-time PCR was conducted using the Biorad CFX96 Quantitative Real-Time PCR instrument. The cycling program for real-time PCR was as follows: 30 s at 95 °C, followed by 40 cycles of 10 s at 95 °C and 20 s at 58 °C. Data were analysed using Biorad CFX Maestro 2.2 software (Biorad). Melting curves for each gene are visible in Supplementary Fig. 2.

### Evaluation of hAMSC senescence

To assess hAMSC senescence the Cell Event Senescence Green Detection Kit (Thermo Fisher Scientific, #C10851) was employed following the manufacturer’s instructions. Briefly, hAMSC were seeded onto specific plastic supports for immunofluorescence analysis (IBIDI, Gräfelfing, Germany, # 81816) at a density of 2.5 × 10^4^ cells/ cm^2^. After exposure to increasing concentrations of BPA for 3 and 24 h, cells were washed once with PBS 1× and fixed in 4% formalin for 10 min. Subsequently, a single wash with PBS 1× was performed, followed by the addition of 100 μL/well of the colorimetric substrate for β-galactosidase (5-bromo-4-chloro-3-indolyl-β-D-galactopyranoside, X-gal) (Merck, Burlington, Massachusetts, USA, #B4252) (diluted 1:100 in wash buffer) in the dark for 2 h at 37 °C in normoxia. After the incubation cells were washed once with PBS 1X, and immunofluorescence signals were detected using MICA platform from Leica. A minimum of 100 cells per field for a total of 3 fields, where this was not possible at least 5 fields, were examined to ensure accurate quantification of β-galactosidase activity after treatment. In addition to cell counting, fluorescence intensity variation following BPA treatment was assessed using ImageJ software. For each image, the ROI was selected based on the cell area, and the mean fluorescence intensity was measured. Background fluorescence was subtracted from each measurement, and the data were reported as normalized integrated density. This quantification allowed for a standardized comparison of p21 expression levels across different experimental conditions.

### Immunofluorescence

hAMSC were seeded onto specific plastic supports for immunofluorescence analysis (IBIDI, Gräfelfing, Germany, #81816) at a density of 2.5 × 10^4^ cells/cm^2^. Following 24 h of exposure to increasing concentrations of BPA, cells were rinsed once with PBS 1× and fixed in 4% formalin for 10 min. Subsequently, cells were washed three times with Tris Buffered Saline (TBS) for 5 min each.

hAMSC were incubated with p21 primary antibody (Waf1/Cip1/CDKN1A p21, sc-6246, Santa Cruz Biotechnology, Texas, USA), diluted 1:100 in normal goat serum (NGS, Invitrogen, Waltham USA, #10000C) and incubated overnight at 4 °C in the dark. Following incubation, cells were washed three times in TBS for 5 min each, incubated for 30 min at 37 °C with secondary antibody (VectorLab DyLight 594, USA, ZH1213) at a concentration of 5 µg/mL. hAMSC were then washed again for three times with TBS, and DAPI (0.1 g/mL, Invitrogen, Waltham, USA, #956) was added for 4 min at 4 °C in the dark. After one final wash with PBS, images were captured using MICA platform from Leica. A minimum of 200 cells across three fields were analysed for most conditions. However, due to a significant reduction of countable cells at the highest BPA concentrations, we increased the number of fields to ensure an accurate quantification of p21 expression after BPA treatment. In addition to cell counting, fluorescence intensity variation following BPA treatment was assessed using ImageJ software. For each image, the ROI was selected based on the cell area, and the mean fluorescence intensity was measured. Background fluorescence was subtracted from each measurement, and the data were reported as normalized integrated density. This quantification allowed for a standardized comparison of p21 expression levels across different experimental conditions.

### ELISA assay

Supernatants obtained from hAMSC exposed to increasing concentrations of BPA for 24 h, with or without an additional treatment of adenosine triphosphate (ATP) for 1.5 h (1.5 mM), were harvested and stored at −80 °C for subsequent analysis. The content of interleukin-1β (IL-1β) was assessed using an enzyme-linked immunosorbent assay (ELISA) kit (BD OptEIA, Franklin Lakes, New Jersey, USA, # 557953) according to the manufacturer’s instructions. Briefly, 96-well flat-bottom plates (Merck, #MSEHNFX40) were coated with 100 μL/well of Coating Antibody and left to incubate for a minimum of 18 h at 4 °C. Afterward, a single wash with Wash Buffer (100 μL/well) was performed, followed by the addition of 200 μL/well of blocking solution (Assay Buffer) for 1 h at room temperature (RT). Subsequently, samples or standards (50 μL/well) were added to the appropriate wells, along with 50 μL/well of Detection Antibody for IL-1β, followed by a 2-h incubation at 4 °C. Five washes were then conducted, and streptavidin-horseradish peroxidase (HRP) solution was added to each well (100 μL/well) and incubated for 30 min at RT. An additional 5 washes were performed with Wash Buffer, and 100 µl/well of 3,3’,5,5’-tetramethylbenzidine (TMB) substrate was added for 30 min at RT. Finally, 50 μL/well of Stop Solution was added, and the absorbance was measured at 450 nm using VictorTM X4 (Perkin Elmer).

### Statistical analysis

Data are represented as mean ± standard deviation (SD) in comparison to the control condition (MetOH). Statistical comparisons were performed using a two-way analysis of variance (ANOVA), followed by a Tukey multiple comparison test for post-analysis. The data represent a minimum of three experiments, with the number of replicates (n value) specified in each Fig. legend. Statistical analysis was conducted using GraphPad Prism 9 software (GraphPad Software, La Jolla, CA, USA), considering a *p*-value less than 0.05 as statistically significant.

## Supplementary information

Supplementary Material

SupplementaryTable 1

## Data Availability

The datasets generated and analysed during the current study are available from the corresponding author on reasonable request.
