# Supplementary material for: Bisphenol-A disrupts mitochondrial functionality leading to senescence and apoptosis in human amniotic mesenchymal stromal cells
Source: Cell Death Discov. 2025 Jul 16;11:327. doi: 10.1038/s41420-025-02620-8 (PMC12267626; doi:10.1038/s41420-025-02620-8)
Supplement: Supplementary file 1 — Supplementary Material [file 41420_2025_2620_MOESM1_ESM.docx]

**Supplementary Figures**

**Bisphenol A Disrupts Mitochondrial Functionality Leading to Senescence and Apoptosis in Human Amniotic Mesenchymal Stromal Cells**

**Running title: Bisphenol A alters cell function**

Sara Ficai^1^*, Andrea Papait^1,2^*, Marta Magatti^3^, Alice Masserdotti^1^, Michael Gasik^4^, Antonietta Rosa Silini^3^, Ornella Parolini^1,2#^

^1^ Department of Life Science and Public Health, Università Cattolica del Sacro Cuore, Rome, Italy,

^2^ Fondazione Policlinico Universitario "Agostino Gemelli" IRCCS, Rome, Italy,

^3^ Centro di Ricerca E. Menni, Fondazione Poliambulanza Istituto Ospedaliero, Brescia, Italy.

4 Seqvera Ltd., Helsinki, Finland

* The authors contributed equally to this work.

^#^ Corresponding author: Ornella Parolini

ornella.parolini@unicatt.it

**
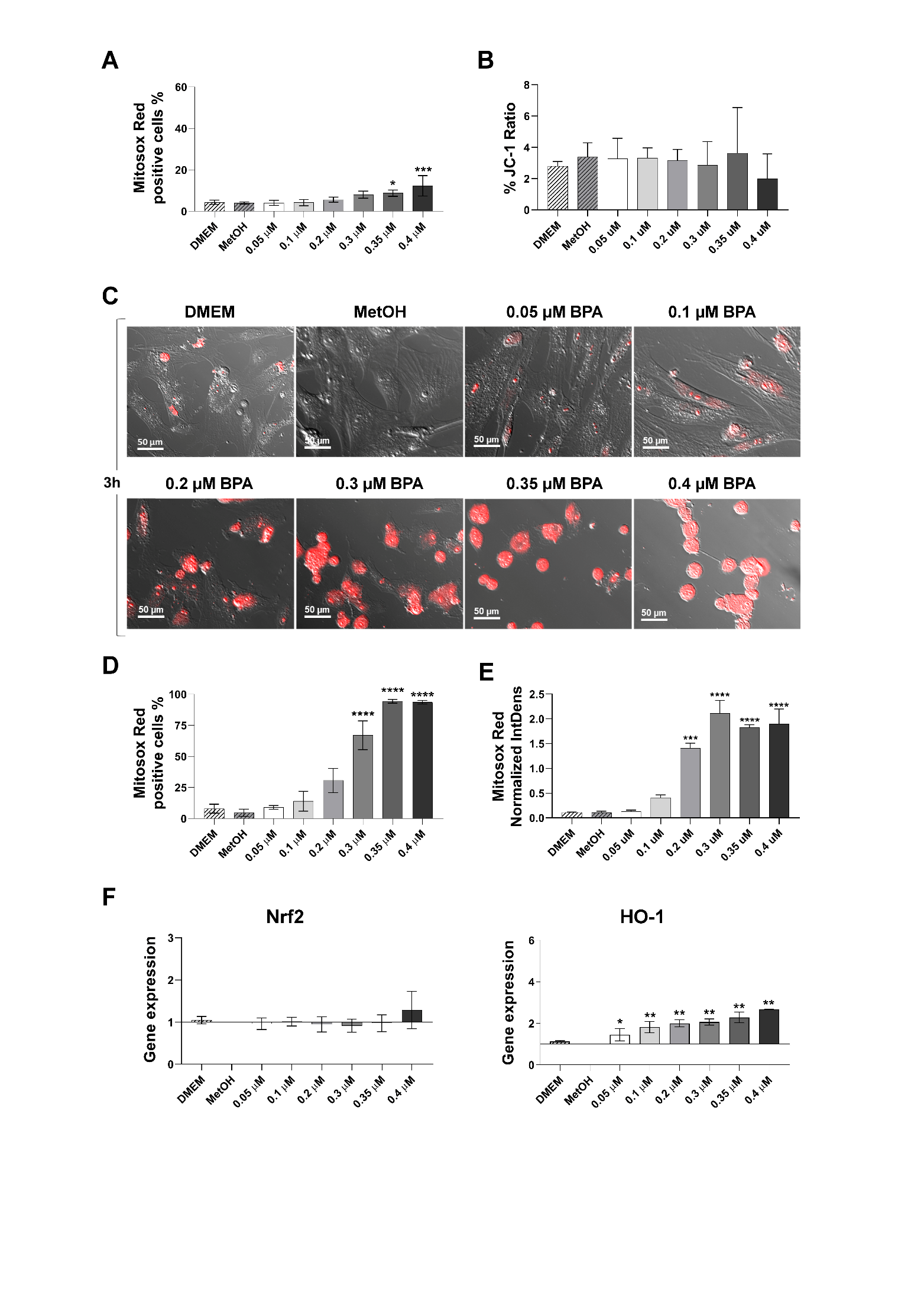
Supplementary Fig. 1**

**Figure S.1 Evaluation of hAMSC oxidative stress after 3 hours of exposure to increasing BPA concentrations.** MtROS production in hAMSC was quantified in flow cytometry and in immunofluorescence using the MitoSOX Red fluorescent dye, after 3 hours of exposure to increasing concentrations of BPA (0.05, 0.1, 0.2, 0.3, 0.35, and 0.4 μM). Results acquired in flow cytometry are presented as the percentage of MitoSOX Red-positive cells (A). Mitochondrial membrane potential (Δψm) variation was assessed using the JC-1 probe and representing the ratio, indicative of mitochondrial depolarization, following BPA exposure (B). Immunofluorescence images of hAMSC after 24 hours of BPA exposure show MitoSOX Red-positive cells (red signal) overlaid on bright-field images (C), acquired at 20× magnification (scale bar, 50 μm). The total number of MitoSOX Red-positive cells was quantified and is reported in (D). Fluorescence intensity of MitoSOX Red, measured as Normalized Integrated Density, is presented in Figure 2E. The antioxidant response of hAMSC was evaluated by quantifying Nrf2 and HO-1 gene expression after 24 hours of BPA exposure, expressed as fold-change relative to the control condition (MetOH) (F).

Histograms represent mean ± SD from n = 4 independent experiments. Statistical analysis was performed versus the control condition: p < 0.01 (*), p < 0.001 (**), p < 0.0001 (****).

**Supplementary Fig. 2**

**
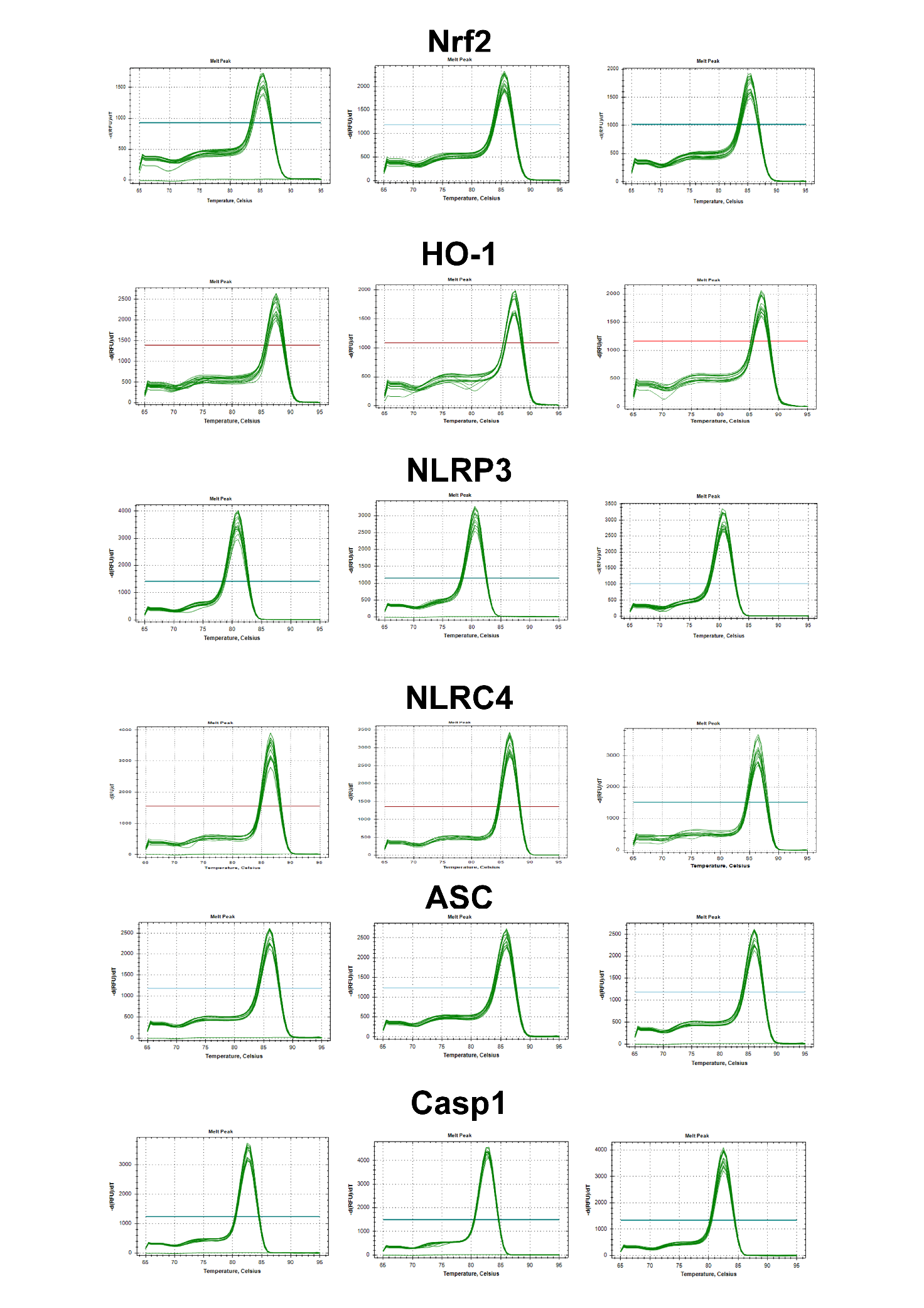
**

**
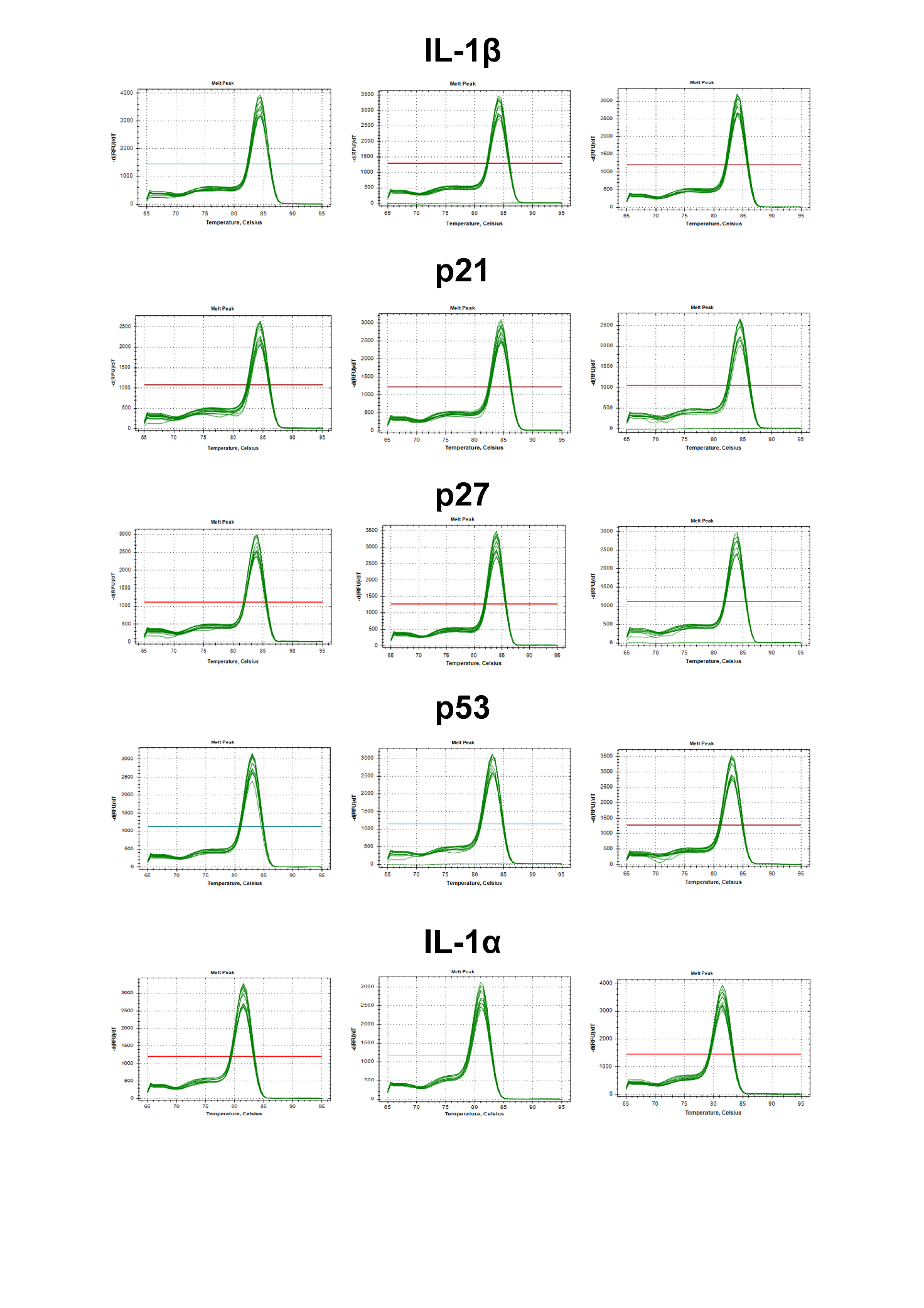
**

**
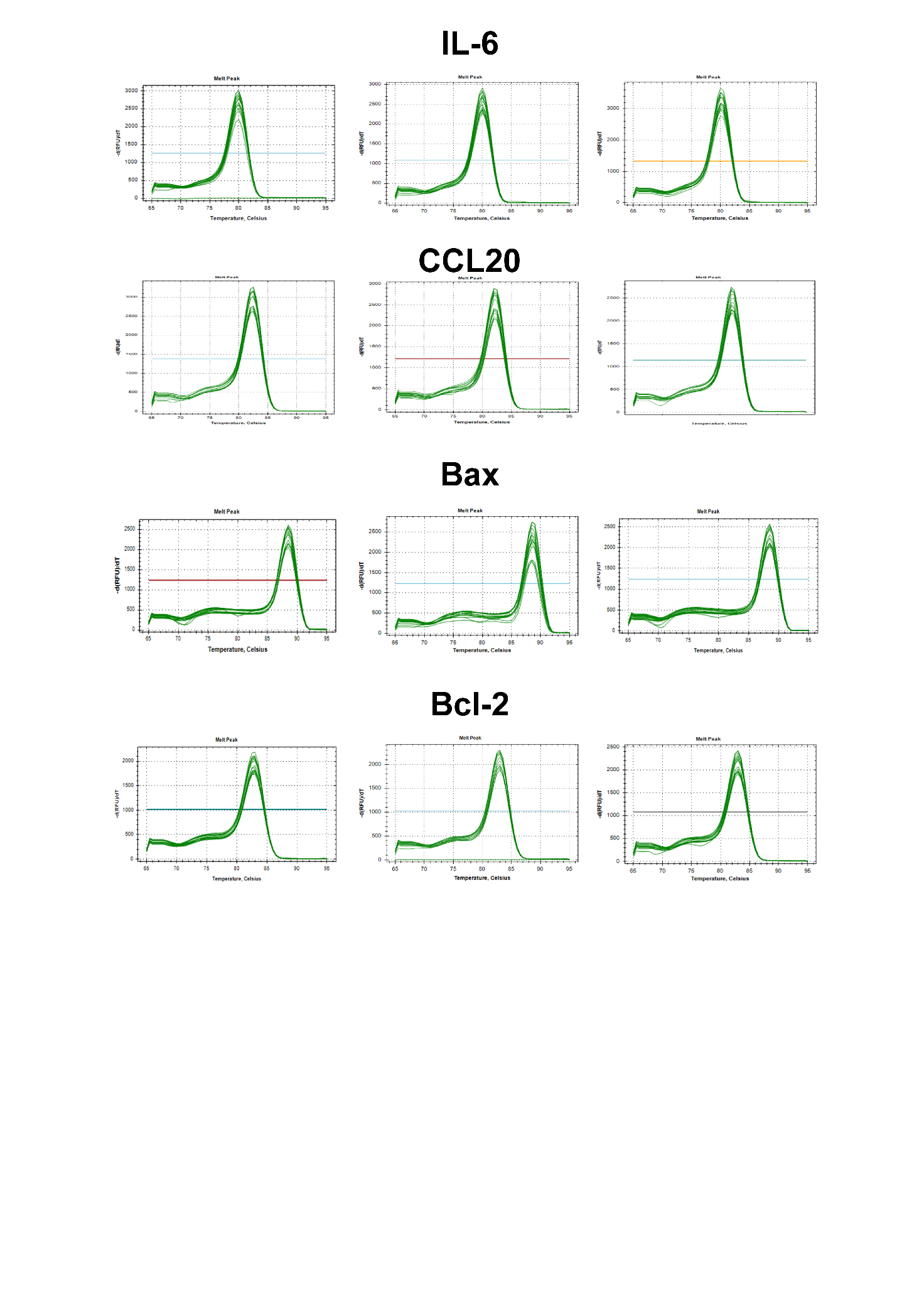
**

**Figure S.2 Melting curve profiles from qRT-PCR analyses**

Melting curve analyses were performed for all quantitative real-time PCR (qRT-PCR) assays to assess the specificity of amplification. The presented curves demonstrate single, sharp peaks, indicative of specific amplification products without primer-dimer formations or nonspecific amplifications.
